# Seropositivity of main vector-borne pathogens in dogs across Europe

**DOI:** 10.1186/s13071-022-05316-5

**Published:** 2022-06-06

**Authors:** Guadalupe Miró, Ian Wright, Helen Michael, Wade Burton, Evan Hegarty, Jaume Rodón, Jesse Buch, Nikola Pantchev, Georg von Samson-Himmelstjerna

**Affiliations:** 1 Department of Animal Health, Veterinary Faculty, Universidad Complutense de Madrid, Madrid, Spain; 2 Mount Veterinary Practice, Fleetwood, UK; 3 IDEXX Laboratories, Inc, Westbrook, ME USA; 4 Institute for Parasitology and Tropical Veterinary Medicine, Freie Universität Berlin, Berlin, Germany

**Keywords:** Leishmania, Dirofilaria immitis, Anaplasma, Ehrlichia, Borrelia burgdorferi, Dogs, Europe, Seropositivity

## Abstract

**Background:**

Canine vector-borne disease (CVBD) has been an area of increasing interest in Europe over the last few decades, and there have been changes in the prevalence and distribution of many of these diseases. Monitoring CVBD infections in Europe is often done by individual countries, but aggregated data for the European countries are helpful to understand the distribution of CVBDs.

**Methods:**

We used an extensive retrospective database of results from point-of-care rapid enzyme-linked immunosorbent assay (ELISA) tests on dogs across Europe to identify distribution and seropositivity in animals tested for selected CVBDs (*Anaplasma* spp.*, Ehrlichia* spp.*, Borrelia burgdorferi, Leishmania* spp., and *Dirofilaria immitis*) from 2016 through 2020. Geographic distribution of positive tests and relative percent positive values were mapped by the Nomenclature of Territorial Units for Statistics classification for regions with sufficient test results for reporting.

**Results:**

A total of 404,617 samples corresponding to 1,134,648 canine results were available from dogs tested in 35 countries over the 5-year study period. Over this period the number of test results per year increased whereas test positivity decreased. *Leishmania* spp. had the largest increase in total test results from 25,000 results in 2016 to over 60,000 results in 2020. Test positivity for *Leishmania* spp. fell from 13.9% in 2016 to 9.4% in 2020. Test positivity fell for *Anaplasma* spp. (7.3 to 5.3%), *Ehrlichia* spp. (4.3 to 3.4%), and *Borrelia burgdorferi* (3.3 to 2.4%). *Dirofilaria immitis* test positivity trended down with a high of 2.7% in 2016 and low of 1.8% in 2018. *Leishmania* spp. test positivity was highest in endemic areas and in several non-endemic countries with low numbers of test results. Co-positivity rates were significantly higher than expected for all pathogen test positive pairs except for *Ehrlichia* spp. with *Borrelia burgdorferi* and *D. immitis* with *Borrelia burgdorferi*.

**Conclusions:**

This study represents the largest data set on CVBD seropositivity in Europe to date. The increase in the number of test results and decreasing test positivity over the study period may reflect changes in testing behavior and increased screening of healthy animals. The Europe-wide mapping of CVBD provides expected test positivity that can help inform veterinarians’ decisions on screening and improve prevention and identification of these important, sometimes zoonotic, diseases.

**Graphical Abstract:**

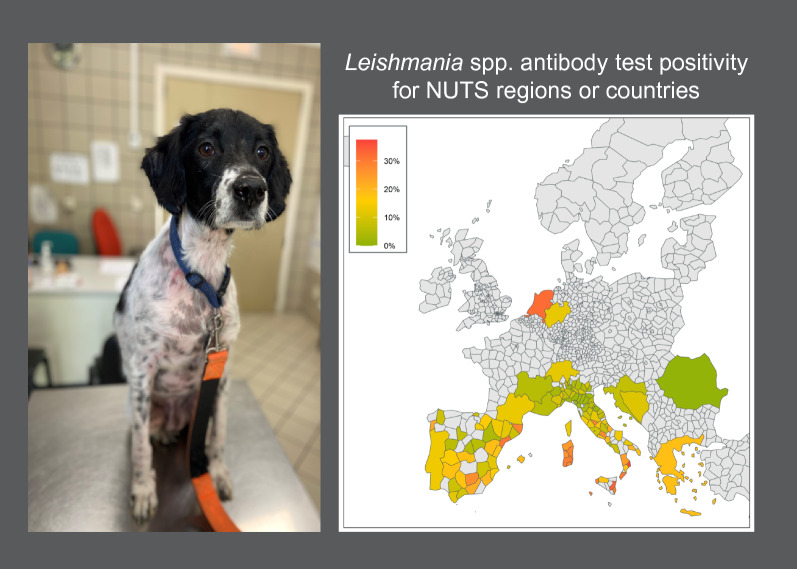

## Background

Canine vector-borne disease (CVBD) has been an area of increasing interest in Europe over the last few decades, and there have been changes in the prevalence and distribution of many of these diseases [1–6]. Many CVBDs (including *Borrelia burgdorferi* complex, *Anaplasma phagocytophilum* and *Anaplasma platys*, *Ehrlichia canis* and *Ehrlichia ewingii*, *Dirofilaria immitis* and *Dirofilaria repens*, and *Leishmania infantum)* have been reported to infect both humans and dogs [7–9]. For *Leishmania infantum* and *Dirofilaria.* spp., dogs are an essential reservoir species for the parasites in endemic areas.

The distribution of CVBDs across Europe has also shifted over recent decades. Changes to the climate and land use have affected the ranges and population size of many insect and tick vectors, and wildlife reservoirs (e.g. rodents, tick transport hosts, migratory birds, wolves, jackals, foxes, wildcats, etc.) [10–21]. Additionally, tourism, travel with dogs and importation of dogs from endemic areas have contributed to introduction of CVBDs to new areas [10, 11]. The range for *Ixodes ricinus*, the primary European tick vector for *Borrelia burgdorferi, Anaplasma* spp., and the tick-borne encephalitis virus, has expanded to northern regions and to higher elevations resulting in increased infections in those areas [12, 20, 22–24]. Another example is *Rhipicephalus sanguineus* (vector for multiple CVBDs), which is being introduced to non-endemic countries (i.e. Germany and Poland) and can establish temporary or permanent populations associated with human habitation or in the environment [12, 17, 25–29]. Increased *D. immitis* infections have been caused by range expansion of both native mosquitos and imported *Aedes* species, climatic changes, and importation of dogs from endemic areas [4, 5, 10, 11, 30]. Canine *L. infantum* infections in northern Europe are thus far almost all related to the importation of infected animals from, or travel to, endemic areas. Uncommon autochthonous non-vector spread has been reported in non-endemic areas, including Germany, UK, Hungary, and Finland [3, 31–34]. Vertical or horizontal transmission through blood transfusion or mating is the most common cause for autochthonous infections in non-endemic areas [1, 3, 10, 34, 35], but rare cases of unexplained horizontal transmission have been reported [31]. Recently, *Phlebotomus* genus sand flies have been identified in regions of northern Europe where they had not previously been found and could potentially result in local transmission of *Leishmania* parasites under the right conditions [33, 36, 37] but the competence of these vectors for transmitting *L. infantum* is still questionable [38]. Climate change may lead to *L. infantum* infections decreasing in some currently endemic areas of southern Europe as they become too hot and dry for *Phlebotomus* sand flies [39].

Monitoring CVBD infections in Europe is often done on a regional level in individual countries, but aggregated data for the European countries can be helpful in understanding the distribution of CVBDs on a broader scale. We used an extensive retrospective database of results from point-of-care rapid enzyme-linked immunosorbent assay (ELISA) tests on dogs across Europe to identify distribution and seropositivity in animals tested for selected VBDs from 2016 through 2020.

## Methods

### Source of data

Results from 2016 to 2020 were generated using point-of-care test kits (IDEXX Laboratories, Inc.) and included: SNAP^®^ 4Dx^®^ Plus Test kit, an in-clinic enzyme-linked immunosorbent assay (ELISA) for detection of *D. immitis* antigen and canine antibodies to *B. burgdorferi*, *Ehrlichia* spp. (*E. canis, E. ewingii*), and *Anaplasma* spp. (*A. phagocytophilum* and *A. platys*); SNAP^®^ HW RT Test kit, an in-clinic ELISA for the detection of *D. immitis* antigen; SNAP^®^ Leishmania Test kit, an in-clinic ELISA for the detection of antibodies to *L. infantum*. These SNAP^®^ tests can be run on serum, plasma, or whole blood. The sample used in individual patients was not captured. The performance of each test has been reported previously [40–43].

Test results were collated directly from veterinary practices testing patients in their clinic (SNAP^®^ Heartworm RT Test, SNAP^®^ Leishmania Test, and SNAP^®^ 4Dx^®^ Plus Test). Test results were stored in IDEXX VetLab^®^ Instrumentation and Software and were entered automatically by the IDEXX SnapShot Dx^®^ Instrument or SNAP Pro^®^ Analyzer or manually by clinic staff. All sample results were obtained from practicing veterinarians in the course of their regular care of the dogs with the consent of the animal owner. To ensure data privacy, results were collected without owner information or canine patient identification; thus, repeat testing events or translocated dogs (i.e. dogs with a travel history to another region) could not be identified or omitted. Similarly, no data were collected about the reason for CVBD testing or about vaccination or prophylaxis usage. All results from veterinary clinics on the European continent and from associated overseas territories owned by European countries were included in the analysis.

### Data analysis

Data analysis and mapping were done using R version 4.0.4 and various R packages [44].^,^^1^^,^^2^^,^^3^

Test positive percentages are reported with 95% confidence intervals calculated using the binomial exact method. Specific tests for differences were not conducted because the intention of this study was to describe pathogen test positivity, not to test hypotheses about differences in test positivity. Positive percentages were presented in tables at country level only if the country had at least 135 results. This threshold was set to ensure precision in estimates.

Co-positivity percentages were estimated for each pair of infections as the percentage of samples that tested positive for multiple pathogens out of all samples that were tested for the respective pathogens. A series of chi-square tests of independence were conducted to determine whether the percentage of co-positives was higher than expected because of chance alone using an alpha of 0.05. *P*-values from the ten pairwise comparisons were adjusted for multiple comparisons using the Holm-Bonferroni method.

### Generation of regional test positivity maps

Mapping of test positivity was done using the Nomenclature of Territorial Units for Statistics (NUTS) classification for Europe.^4^ NUTS classifications have different levels of division within each European country [45]. NUTS 0 represents the boundaries of the country. NUTS 1 represents large regions within a country. NUTS 1 regions are further subdivided into NUTS 2 level regions and then further subdivided into NUTS 3. Each result was assigned to its NUTS 0 (country level) through NUTS 3 units for analysis.

The preference was to display data at the smallest appropriate NUTS level for each region. To balance the desire to provide meaningful granular regional data with unequal distribution of data across different regions, the following system was used to determine which NUTS level would be displayed. First, a minimum of three clinics with at least one result each was required within each NUTS region to qualify for inclusion in the display at that NUTS level. This restriction was included to ensure privacy of the clinics. Second, at least 50% of the smaller NUTS regions within an individual larger NUTS region had to qualify for display (at least 3 clinics and at least 135 total results for the region) at the smaller NUTS region level. If < 50% of the smaller regions qualified for display, the corresponding next larger region was evaluated for inclusion. Mapping of *Leishmania* spp. positives in France and Germany was conducted at NUTS level 1, even though < 50% of these regions qualified for display. This was done because most tests in these countries were from a few geographical areas, and very few tests were from outside of these areas. To present these limited data at the NUTS level 0 (country level) was considered to be misrepresentative.

For regions that qualified for inclusion, test positivity rate over the 5-year study period was displayed on a gradient from green (lower test positivity) to red (higher test positivity). Regions that did not qualify for inclusion are colored pale gray. NUTS classification is not available in Russia. Russia was not included in the maps because of the low number of samples, which were not considered representative of CVBD. The Canary Islands of Spain were included in the map for *D. immitis* because they have been previously found to be hyperendemic for these infections (prevalence of 58%) since 1995 with an important decrease in the last decades (prevalence around 18%) [46, 47] and because travel to island territories with pets can spread disease.

## Results

### Summary

**Fig. 1:**
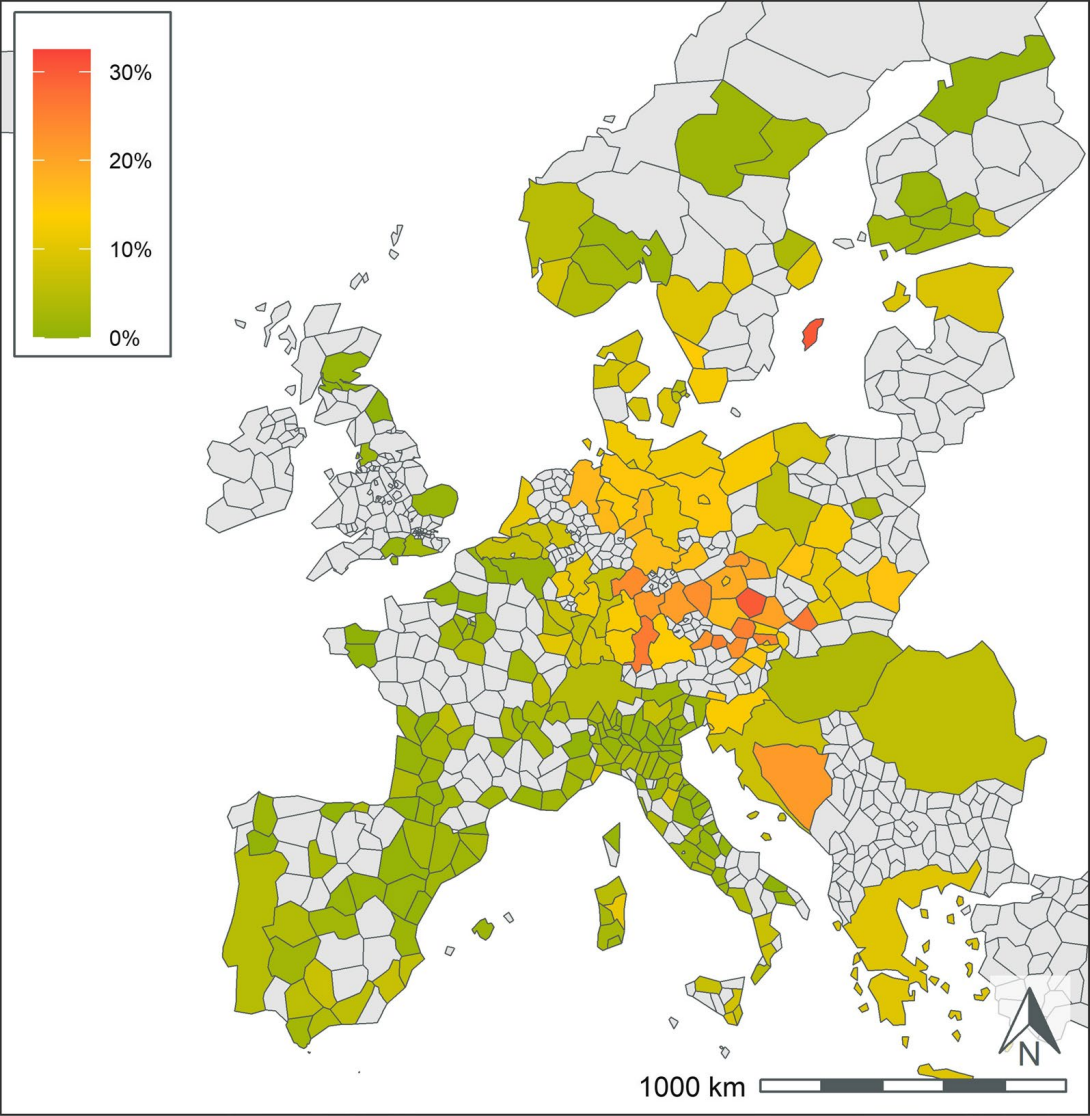
*Anaplasma* spp. antibody test positivity for NUTS regions or country over the study period (2016–2020. NUTS levels are shown with the most geographic detail allowed by the regional data. Gray regions did not have sufficient results for evaluation of region-specific test positivity analysis

**Fig. 2:**
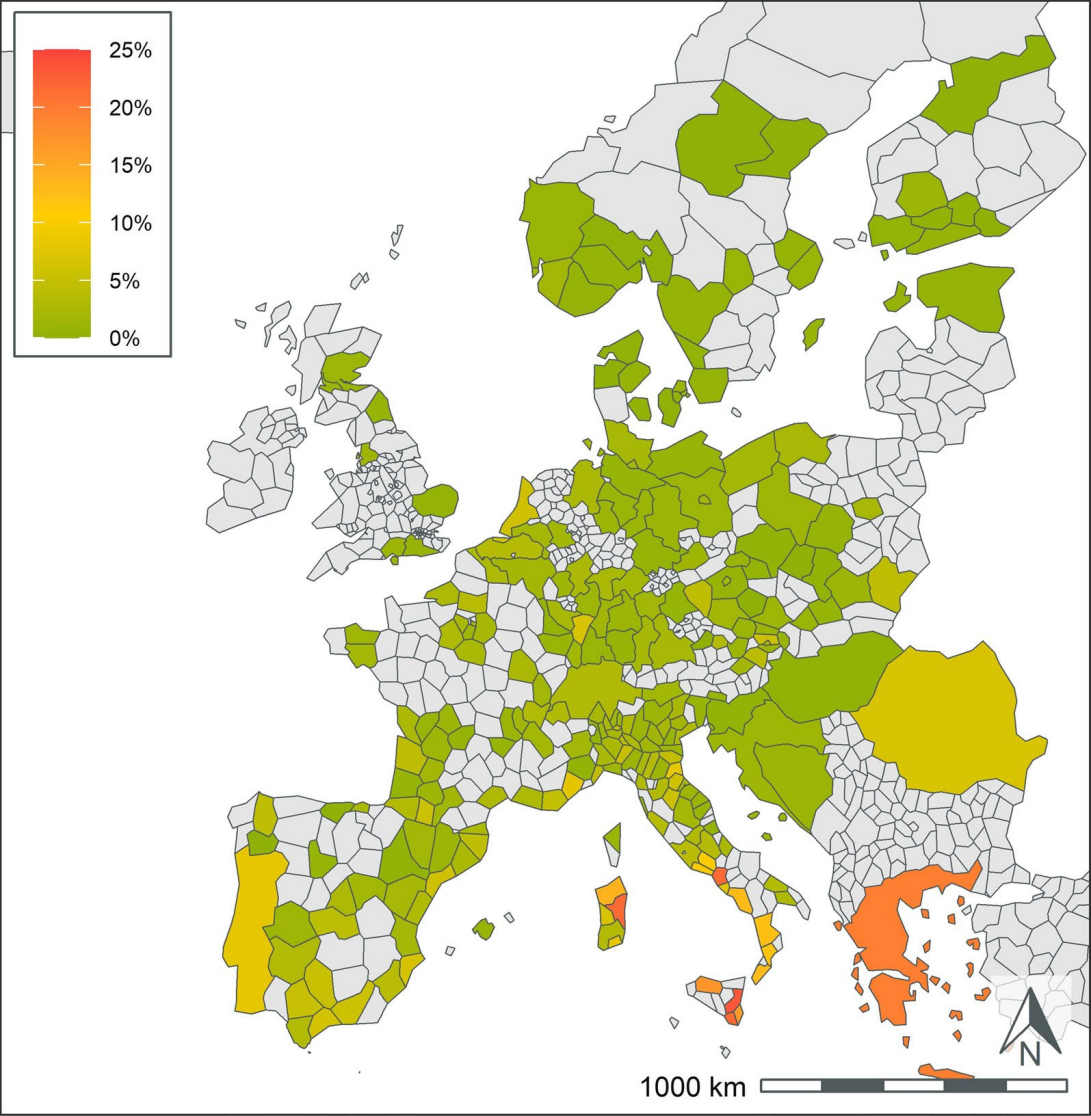
*Ehrlichia* spp. antibody test positivity for NUTS regions over the study period (2016–2020). NUTS levels are shown with the most geographic detail allowed by the regional data. Gray regions did not have sufficient results for evaluation of region-specific test positivity analysis

**Fig. 3:**
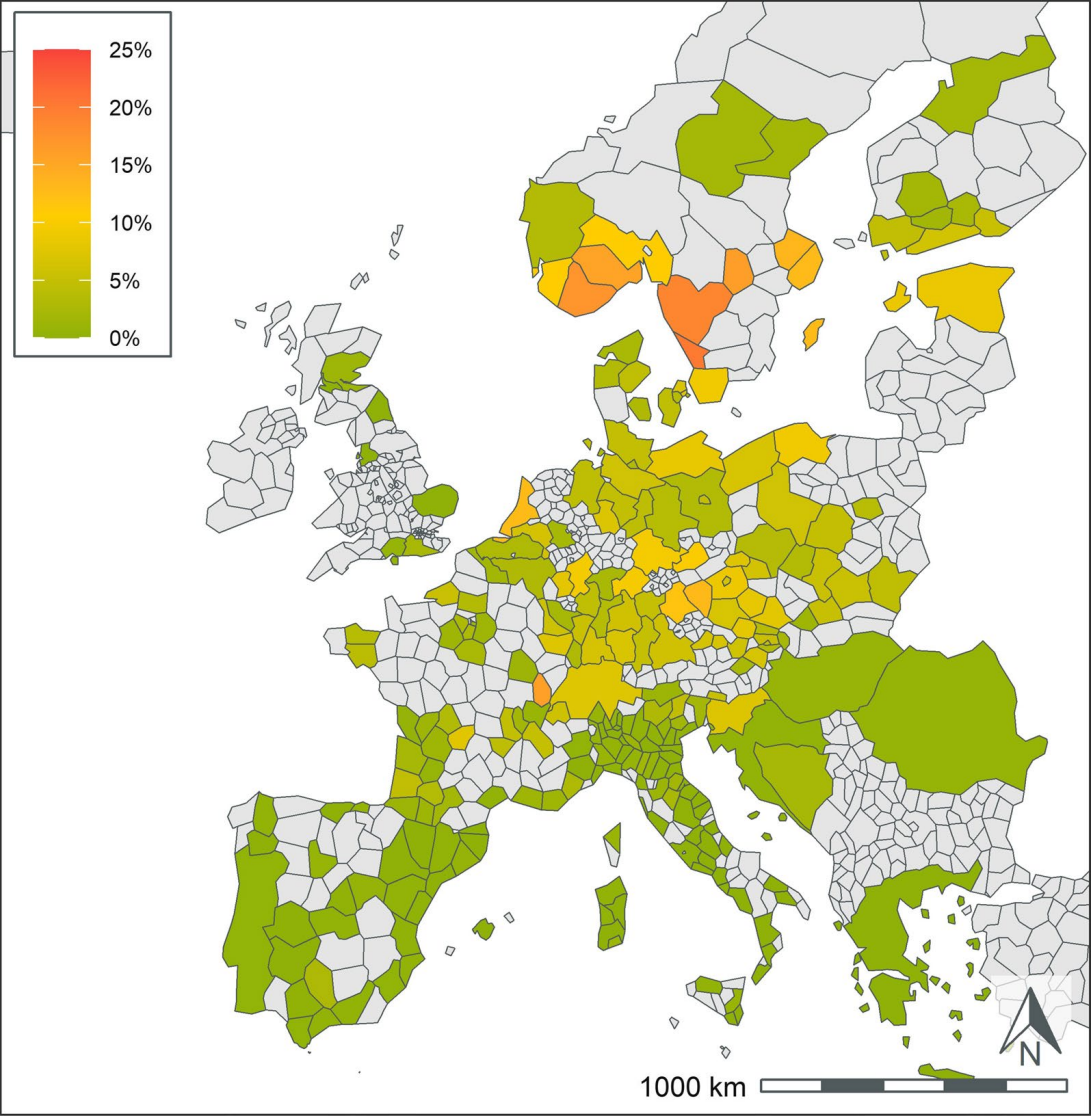
*Borrelia burgdorferi* antibody test positivity for NUTS regions or country over the study period (2016–2020). NUTS levels are shown with the most geographic detail allowed by the regional data. Gray regions did not have sufficient results for evaluation of region-specific test positivity analysis

**Fig. 4:**
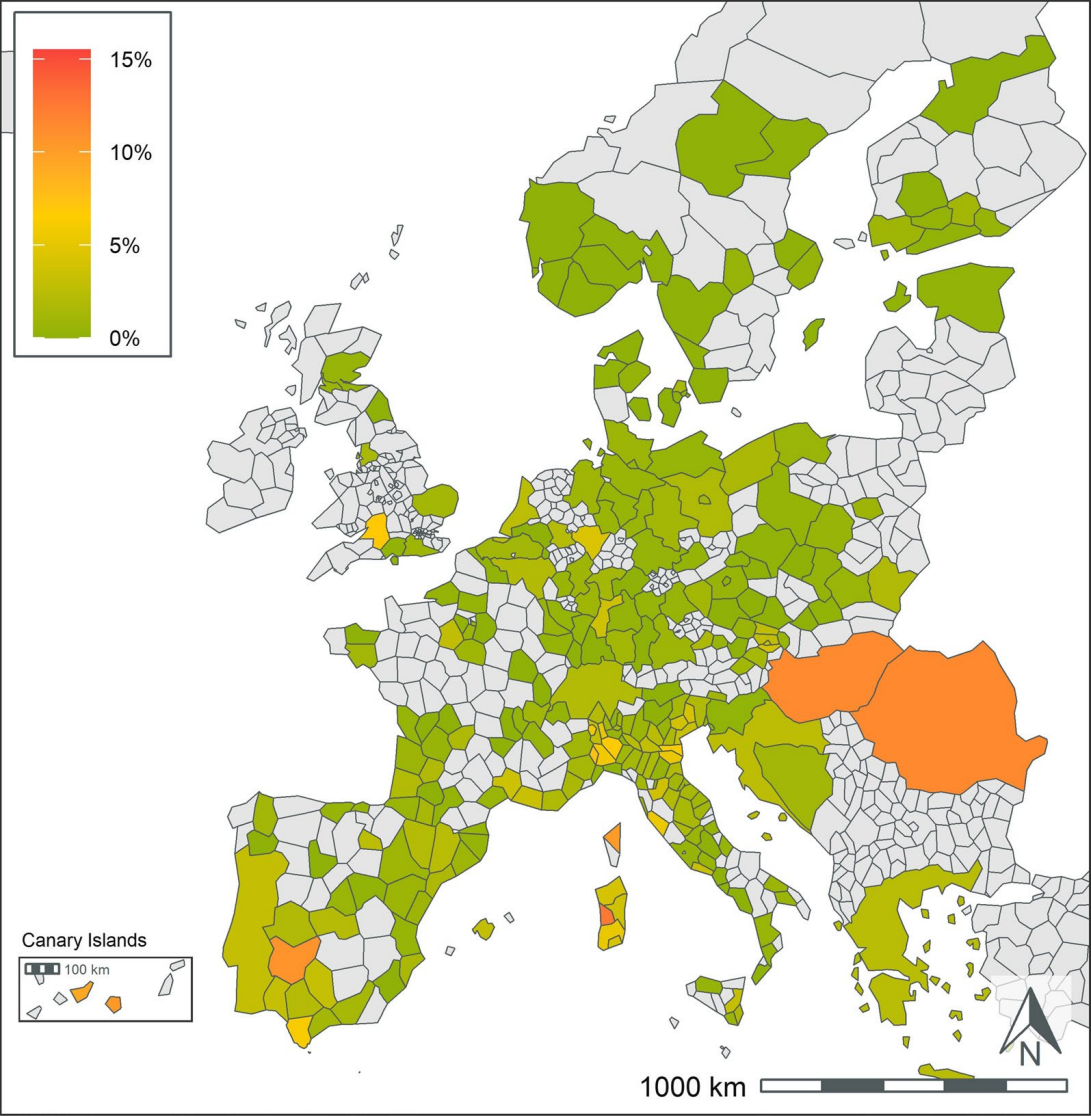
*Dirofilaria immitis* antigen test positivity for NUTS regions or country over the study period (2016–2020). NUTS levels are shown with the most geographic detail allowed by the regional data. Gray regions did not have sufficient results for evaluation of region-specific test positivity analysis. Test positivity in the Canary Islands (a high endemic area) was added to the figure (not to scale) for reference

**Fig. 5:**
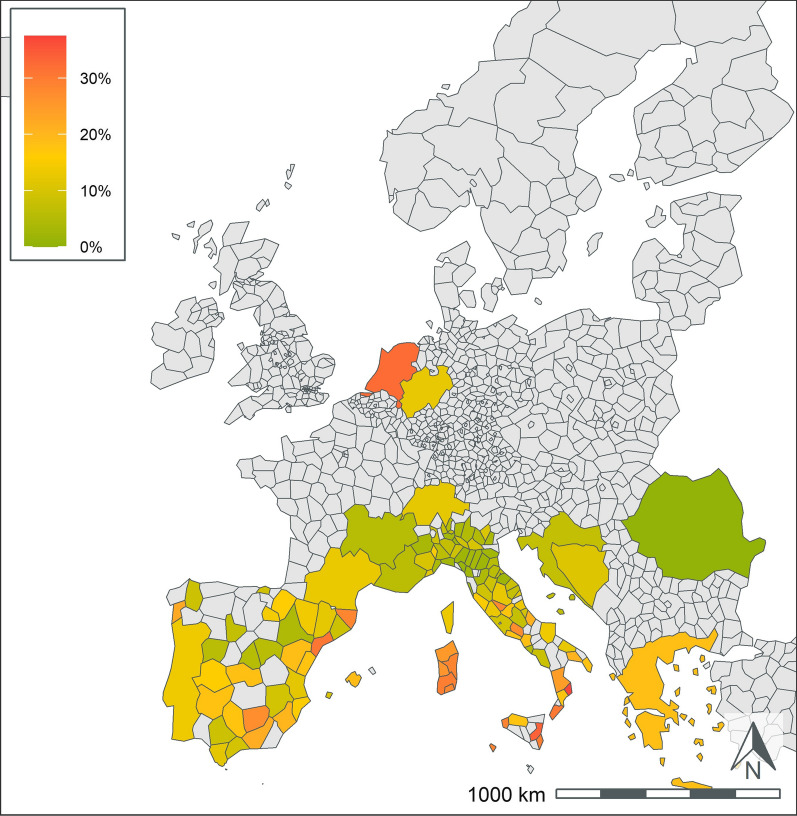
*Leishmania* spp. antibody test positivity for NUTS regions or country over the study period (2016*–*2020). NUTS levels are shown with the most geographic detail allowed by the regional data. Gray regions did not have sufficient results for evaluation of region-specific test positivity analysis. Results from Germany and France are shown at NUTS level 1 since test results were restricted to a few areas within the country

**Fig. 6:**
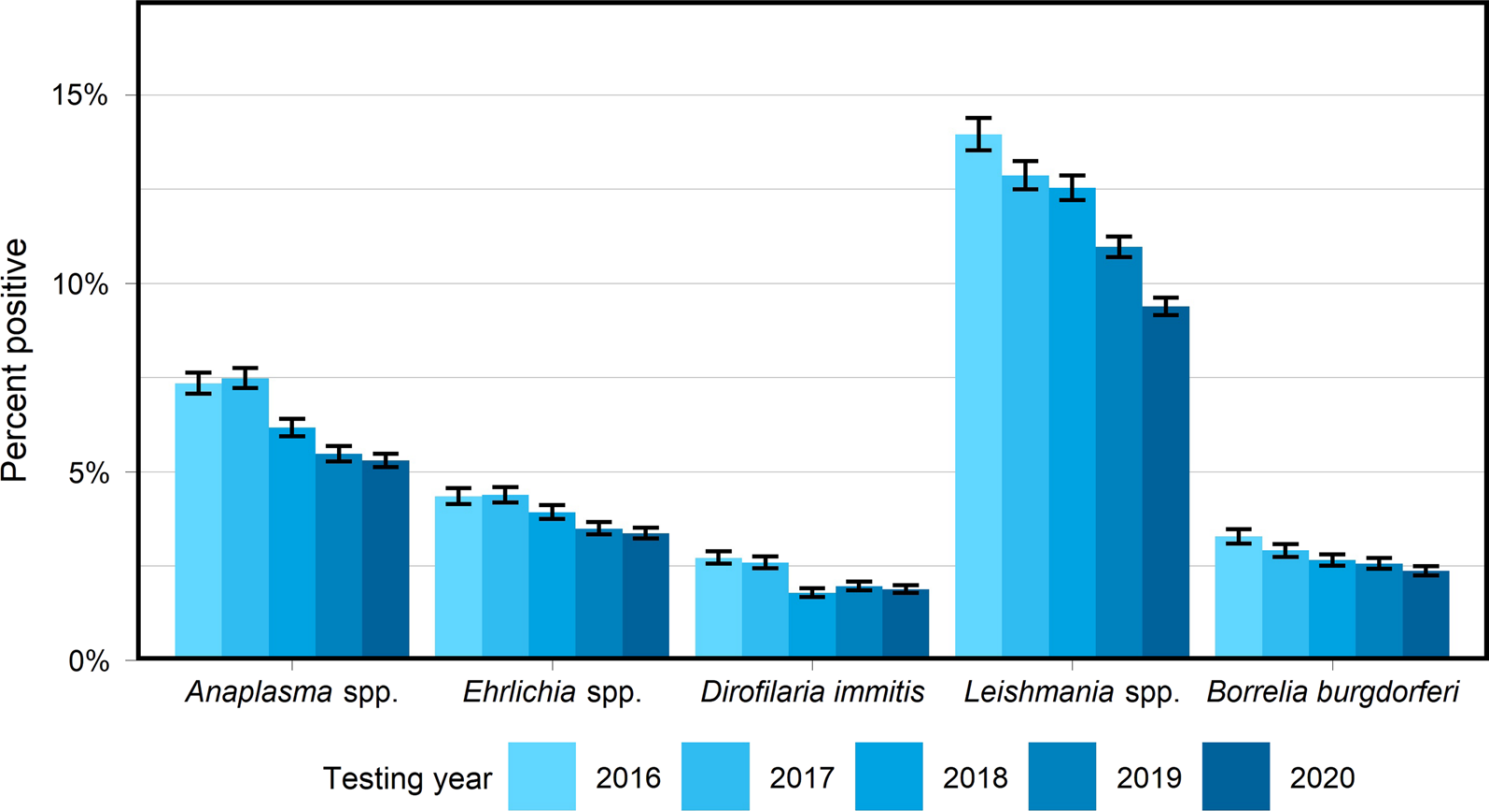
Yearly European test positivity for each pathogen. Positive percent of all tests and 95% confidence intervals (bars) are shown for each year. Non-overlapping confidence intervals support significant differences in test positivity between years

A total of 404,617 samples corresponding to 1,134,648 results were available from dogs tested in 26 European countries over the 5-year period summarized in the current paper (Table 1). This represents results from > 251,000 tests for antigen of *D. immitis*, 211,000 tests for antibodies to *L. infantum*, and 224,000 tests for antibodies to each of *B. burgdorferi*, *Ehrlichia* spp., and *Anaplasma* spp. Geographic distribution of positive tests and relative percent positive values are shown by NUTS classification in Figs. 1, 2, 3, 4, 5. Overall, each disease showed a decrease in percent positive results (Fig. 6) as the total tests performed increased (Table 1).

### Anaplasma spp., Ehrlichia spp., and Borrelia burgdorferi

The total number of results for tick-borne diseases in Europe increased each year (Table 1) with a trend of declining rates of test positivity for each pathogen (Fig. 6). Similar trends of declining test positivity were noted for each of the pathogens. Annual European test positivity rates decreased from 7.3% in 2016 to 5.3% in 2020 for *Anaplasma* spp., from 4.3% in 2016 to 3.4% in 2020 for *Ehrlichia* spp., and from 3.3% in 2016 to 2.4% in 2020 for *Borrelia burgdorferi* (Table 2).

*Anaplasma* spp. antibody test positivity rates for the study period varied regionally and were higher (> 10%) in Austria, Bosnia and Herzegovina, Czech Republic, Germany, Poland, Slovakia, Slovenia, and Sweden and lower (< 5%) in Andorra, Belgium, Finland, France, Hungary, Italy, Malta, Norway, Portugal, Russia, Spain, Switzerland, and the UK (Table 3). Remaining countries had test positivity between 5 and 10%. Geographic distribution of positive test results using the NUTS classification is displayed for regions with sufficient test results for reporting (Fig. 1).

*Ehrlichia* spp. antibody test positivity rates were higher (> 3%) in Greece, Italy, Lithuania, The Netherlands, Portugal, Romania, Russia, Spain, and Switzerland and lower (< 1%) in Denmark, Estonia, Finland, Hungary, Norway, Slovakia, Slovenia, and Sweden (Table 3). Remaining countries had test positivity between 1 and 3%. Greece showed the highest percent positive results (19.6%) while Denmark, Estonia, Hungary, and Slovenia all reported < 0.5% positive results during the study period (Fig. 2).

*Borrelia burgdorferi* antibody positivity was concentrated in Northern and Eastern Europe with higher rates of positivity (> 5%) in Austria, Czech Republic, Estonia, Finland, Germany, Lithuania, The Netherlands, Norway, Poland, Slovenia, Sweden, and Switzerland and lowest rates (< 1%) in Andorra, Croatia, Greece, Hungary, Italy, Malta, Portugal, Romania, and Spain (Table 3). Remaining countries had test positivity between 1 and 5%. The highest test positivity was seen in Sweden (13.3%) and lowest in Greece (< 0.1%). Test positivity based on NUTS classification in the EU and UK is presented in Fig. 3.

### Dirofilaria immitis

The total number of *D. immitis* test results in Europe increased steadily over the study period with 1.8 × more tests run in 2020 than in 2016 (Table 1). The yearly percent positive results for *D. Immitis* antigen trended down over the 5-year study period (Fig. 6) with a high of 2.7% in 2016 and low of 1.8% in 2018 (Table 4).

*Dirofilaria immitis* positivity rates varied regionally with > 9% positive tests in Hungary, Romania, and Russia and < 1% positive results in 12 countries, Czech Republic, Denmark, Estonia, Finland, France, Germany, Malta, Norway, Poland, Slovakia, Slovenia, and Sweden (Table 3). Remaining countries had between 1 and 9% test positivity. Malta and Norway each had < 0.1% positive results (Table 3).

### Leishmania spp

*Leishmania* spp. infection showed a similar trend of increasing result numbers each year in Europe and decreasing test positivity. The yearly percent of positive *Leishmania* antibody test results decreased from 13.9% in 2016 to 9.4% in 2020 (Table 2). Substantial year-over-year decreases with non-overlapping confidence intervals suggest significant decreases for each pair of sequential years except 2017–2018 (Fig. 6).

*Leishmania* spp. results were primarily available from endemic areas in southern Europe (Table 3, Fig. 5). There was wide variability in the number of test results from different countries. Italy and Spain each had > 90,000 test results and test positivity of 11.9% and 10.7%, respectively. All other endemic countries had < 10,000 test results. Greece and Malta had the highest rate of test positivity in the endemic countries with 18.5% and 15.9%, respectively. All other endemic areas, other than Romania, had test positivity rates > 7%. Within France, most test results originated from endemic areas in southern regions. Only three non-endemic countries, The Netherlands, Switzerland, and Germany, had sufficient tests to report test positivity. These countries had high test positivity and < 700 test results (Table 3).

### Co-positivity

Co-positivity was evaluated on a Europe-wide scale. Actual co-positivity rates were significantly higher than expected for all pathogen test positive pairs except for *Ehrlichia* spp. and *Borrelia burgdorferi* co-positivity and *D. immitis* and *Borrelia burgdorferi* (Table 4). The overall total rate of co-positives was 2–3 times more than the expected for most pairings. The highest percentage of co-positivity was for *Ehrlichia* spp. and *Leishmania* spp. (1.44%), *Anaplasma* spp. and *Ehrlichia* spp. (0.81%), and *Anaplasma* spp. and *Leishmania* spp. (0.78%).

## Discussion

In this study we used data from point-of-care ELISA testing to map distribution and test positivity of CVBDs across Europe. This is to date the most extensive study done on vector-borne infection in dogs in Europe with > 1.1 million test results over a 5-year period. It provides data about the current state of vector-borne infection in tested dogs but is not an unbiased random sample representing prevalence in all dogs in Europe. Over the study period, the yearly number of tests performed increased by a factor of 1.7–2.5 from 2016 to 2020 for each pathogen, while the percentage of positive test results declined. Testing behavior likely impacted these trends. In this study, we did not have access to clinical information and cannot determine how many dogs were sampled as part of wellness screening or because of clinical illness. Substantial variation exists between veterinarians and countries in whether CVBD testing is performed as part of preventative care or primarily in cases with clinical illness [48, 49]. For instance, perceptions of veterinarians around the prevalence of *Leishmania* in their area of practice is related to their likelihood to test and to prescribe preventative measures to their patients [49, 50]. Substantial differences have previously been demonstrated in test positivity in CVBDs when healthy and clinically suspect dogs are tested [51]. The European Scientific Counsel Companion Animal Parasites (ESCCAP) recommends serologic screening for CVBDs endemic in the animal’s home region and within 3–6 months following travel to areas where other CVBDs are endemic. This bias due to different testing criteria in endemic and non-endemic areas was most notable with *Leishmania*. Although Spain and Germany had similar test positivity, 98,737 test results were available for Spain and only 686 tests for Germany, where testing is primarily performed on dogs with a travel or importation history [52]. Additionally, increasing adoption of screening testing in some regions during the 4-year study period may have impacted the results by identifying infected animals who are not sick and reduced the expected percentage of positive test results [53–56].

In addition to changes in testing behavior over the study period, increased use of preventatives and other efforts to decrease exposure to vectors, and newer treatment modalities may have decreased new exposure of dogs to CVBDs or the likelihood of positive results. For *Leishmania* spp. infections increased usage of insect repellents with proven efficacy to prevent sand fly bites [57, 58] and antiparasite products [59] likely contribute to this decline. Additionally, *Leishmania* test positivity could also have been reduced by long-term reductions in antibody levels of patients treated with domperidone [60, 61] and the use of other immunomodulators [62, 63]. For other CVBDs, the more modest declines may be similarly multi-factorial with changes in screening behavior and preventative use in combination with year-to-year climate effects on vector populations. Information about tick, mosquito, and sandfly populations was not collected as part of this study, so it was not possible to identify how or whether changes in vector populations impacted test positivity. Determining the contribution of potential causal factors in these declines would require additional study.

Differences in the number of test results in a particular region were also reflected in the regional resolution of the maps. The NUTS system was used since it is a convenient system for mapping subregions of European countries that is tied to the population in each subregion. Countries (NUTS 0) are divided, when population size allows, into large regions with 3–7 million residents (NUTS 1), which are subdivided into smaller regions with 800,000–3 million residents (NUTS 2), which are then subdivided again into regions of 150,000–800,000 residents (NUTS 3). Since different regions had different numbers of tests available and the goal of the study was to show clinically meaningful data for as many regions as possible, the granularity of regional data presented varies. Where possible, NUTS 3 classification was used to generate regional maps, but in some instances the results were presented at country level when the number of results did not support finer resolution.

Using these data, it was not possible to draw information about whether there was an expansion of CVBDs into new areas since we cannot identify where dogs were infected by a particular pathogen. Positive test results outside of endemic areas could represent imported cases or expansion of CVBDs into new areas.

This study focused on test positivity from point-of-care testing and did not confirm positive tests. Rarely, false-positive results on the SNAP HW RT tests, but not the SNAP 4Dx Plus test, have been reported in *Angiostrongylus vasorum*-positive dogs [64]. This potential for false positives due to *A. vasorum* infection is not likely to have substantially impacted the data since almost all *D. immitis* results in the data set were from SNAP 4Dx Plus tests. Similar false positives due to other CVBD infections have not been reported for the other tests included in this study. The VlsE C6 peptide (C6) utilized to detect *B. burgdorferi* antibodies does not cross-react with other *Borrelia* spp. or Lyme vaccination (regardless of the vaccine type) [65–68].

Co-positivity was higher than expected by chance for almost all pairs of pathogens. Results are presented in aggregate representing tests from all available European counties. They do not account for differences in regional risk of infection for different CVBDs or identify whether co-positives were higher in some regions. Additionally, since no clinical information was collected, we cannot identify dogs with previous exposure from those that are clinically ill. The number of dogs positive for two pathogens was relatively low, but dogs positive for one pathogen should be tested for other CVBD pathogens. High co-positivity rates for *Leishmania* spp. and *Ehrlichia* spp. and for *Leishmania* spp. and *Anaplasma* spp. have previously been described in dogs in, or imported from, southern Europe [51, 69–73]. For example, dogs imported to Germany showed a significant rate of co-positivity for *Leishmania* spp. and *Ehrlichia* spp. (617 out of 15,955 tested dogs), and to a lesser but still significant extent for *Leishmania* spp. and *Babesia canis, Ehrlichia* spp. and *Babesia canis* and for *Leishmania* spp. and *Anaplasma* spp. [10]. Dogs with clinical illness associated with CVBDs are more likely to have co-positive results for other CVBDs [51, 72, 73] and may have more severe clinical presentations. The cause for the higher than expected co-positivity is not clear, and potential contributing factors for increased co-infection risk may include changes in distribution or life cycle of CVBDs and associated vectors, outdoor dog housing and associated increased exposure to vectors, lack of effective insect repellents or antiparasitic treatments, and immunocompromised status [74–76].

## Conclusions

This study provides CVBD test positivity and geographic test positivity at the most granular scale possible for countries in Europe from 2016 to 2020. During the study period, increasing numbers of test results were available each year even as the proportion of positive tests decreased. The most substantial decline was in *Leishmania* spp. test positivity. Increases in use of effective preventatives and routine screening and preventative care of animals without clinical leishmaniosis also likely play roles in the increasing total number of tests and decreasing test positivity. This study represents the largest data set on test positivity for CVBDs for European countries and can help inform veterinarians on the results in their geography and improve prevention of these important clinical and zoonotic diseases.

**Table 1:** Number of European dog sample tests by year for individual vector-borne diseases

Year	*Anaplasma *spp.	*Ehrlichia *spp.	*Borrelia burgdorferi*	*Dirofilaria immitis*	*Leishmania *spp.
2016	34,657	34,658	34,661	38,126	25,394
2017	38,181	38,181	38,181	41,694	30,947
2018	42,529	42,529	42,533	47,535	39,676
2019	49,733	49,733	49,734	56,325	52,792
2020	58,924	58,924	58,924	67,324	62,753
Total	224,024	224,025	224,033	251,004	211,562

**Table 2:** Percent of European dog sample positive test results and 95% confidence intervals by year for individual vector-borne diseases

Year	*Anaplasma *spp.	*Ehrlichia *spp.	*Borrelia burgdorferi*	*Dirofilaria immitis*	*Leishmania *spp.
Positive % of tests (95% CI)					
2016	7.3% (7.1–7.6%)	4.3% (4.1–4.6%)	3.3% (3.1–3.5%)	2.7% (2.6–2.9%)	13.9% (13.5–14.4%)
2017	7.5% (7.2–7.7%)	4.4% (4.2–4.6%)	2.9% (2.7–3.1%)	2.6% (2.4–2.7%)	12.9% (12.5–13.2%)
2018	6.2% (5.9–6.4%)	3.9% (3.7–4.1%)	2.6% (2.5–2.8%)	1.8% (1.7–1.9%)	12.5% (12.2–12.9%)
2019	5.5% (5.3–5.7%)	3.5% (3.3–3.7%)	2.6% (2.4–2.7%)	2.0% (1.8–2.1%)	11.0% (10.7–11.2%)
2020	5.3% (5.1–5.5%)	3.4% (3.2–3.5%)	2.4% (2.2–2.5%)	1.9% (1.8–2.0%)	9.4% (9.2–9.6%)

**Table 3:** Percent of positive test results, total number of dog samples tested, and 95% confidence intervals for each country and respective vector-borne disease for the 2016–2020 study period

Country	*Anaplasma *spp.	*Ehrlichia *spp.	*Borrelia burgdorferi*	*Dirofilaria immitis*	*Leishmania *spp.
Positive % of tests (95% CI)					
Andorra	2.7% (187; 0.9–6.1%)	2.1% (187; 0.6–5.4%)	0.0% (187; 0.0–2.0%)	2.1% (187; 0.6–5.4%)	–
Austria	17.3% (4572; 16.2–18.4%)	2.5% (4572; 2.0–3.0%)	6.1% (4572; 5.4–6.9%)	1.9% (4578; 1.6–2.4%)	–
Belgium	4.5% (772; 3.2–6.2%)	2.8% (772; 1.8–4.3%)	3.0% (772; 1.9–4.4%)	1.5% (843; 0.8–2.6%)	–
Bosnia and	21.4% (3671;	1.1% (3671;	2.2% (3671;	1.0% (3698;	11.0% (172;
Herzegovina	20.1–22.7%)	0.8–1.5%)	1.7–2.7%)	0.7–1.4%)	6.8–16.7%)
Croatia	7.4% (2417; 6.4–8.5%)	1.4% (2417; 0.9–1.9%)	0.4% (2417; 0.2–0.7%)	2.6% (2417; 2.0–3.3%)	7.0% (1761; 5.9–8.3%)
Czech Republic	19.3% (6238; 18.3–20.3%)	1.5% (6238; 1.2–1.8%)	7.6% (6238; 7.0–8.3%)	0.2% (6244; 0.1–0.4%)	–
Denmark	7.7% (7784; 7.1–8.3%)	0.3% (7784; 0.2–0.5%)	4.4% (7784; 4.0–4.9%)	0.4% (8978; 0.2–0.5%)	–
Estonia	9.1% (451; 6.6–12.1%)	0.4% (451; 0.1–1.6%)	8.6% (451; 6.2–11.6%)	0.2% (451; 0.0–1.2%)	–
Finland	3.5% (6084; 3.0–3.9%)	0.6% (6084; 0.4–0.8%)	5.6% (6084; 5.0–6.2%)	0.4% (6084; 0.2–0.5%)	–
France	2.7% (18,070; 2.5–3.0%)	2.8% (18,070; 2.6–3.1%)	2.7% (18,074; 2.5–3.0%)	0.8% (18,356; 0.7–1.0%)	8.7% (5307; 8.0–9.5%)
Germany	14.2% (20,582; 13.7–14.7%)	1.4% (20,582; 1.2–1.5%)	6.0% (20,583; 5.7–6.3%)	0.8% (20,632; 0.7–1.0%)	11.8% (686; 9.5–14.5%)
Greece	9.9% (6488; 9.1–10.6%)	19.6% (6488; 18.6–20.6%)	0.0% (6488; 0.0–0.1%)	2.4% (6497; 2.1–2.8%)	18.5% (9568; 17.7–19.3%)
Hungary	3.9% (593; 2.5–5.8%)	0.2% (593; 0.0–0.9%)	0.8% (593; 0.3–2.0%)	11.5% (724; 9.2–14.0%)	–
Italy	2.6% (64,879; 2.5–2.7%)	5.1% (64,879; 5.0–5.3%)	0.4% (64,879; 0.4–0.5%)	1.9% (84,105; 1.8–2.0%)	11.9% (90,532; 11.7–12.1%)
Lithuania	8.9% (203; 5.3–13.7%)	5.4% (203; 2.7–9.5%)	11.8% (203; 7.7–17.1%)	5.4% (203; 2.7–9.5%)	–
Malta	0.0% (161; 0.0–2.3%)	11.8% (161; 7.3–17.8%)	0.0% (161; 0.0–2.3%)	0.0% (161; 0.0–2.3%)	15.9% (289; 11.9–20.7%)
The Netherlands	9.1% (1154; 7.5–10.9%)	4.7% (1154; 3.5–6.1%)	9.7% (1154; 8.1–11.6%)	1.8% (1170; 1.1–2.7%)	32.4% (136; 24.6–0.9%)
Norway	3.5% (3051; 2.9–4.2%)	0.7% (3051; 0.4–1.0%)	10.2% (3051; 9.1–11.3%)	0.0% (3051; 0.0–0.2%)	–
Poland	10.5% (3812; 9.6–11.5%)	1.2% (3812; 0.9–1.6%)	5.4% (3812; 4.7–6.1%)	0.8% (3816; 0.6–1.2%)	–
Portugal	4.7% (1285; 3.7–6.1%)	8.2% (1285; 6.7–9.8%)	0.2% (1285; 0.0–0.7%)	3.1% (1690; 2.3–4.0%)	13.8% (1329; 12.0–15.7%)
Romania	5.9% (13,995; 5.6–6.3%)	7.0% (13,995; 6.5–7.4%)	0.6% (13,995; 0.5–0.8%)	11.5% (14,169; 11.0–12.1%)	0.6% (2546; 0.3–1.0%)
Russia	4.7% (1819; 3.7–5.7%)	10.9% (1819; 9.5–12.4%)	2.1% (1819; 1.5–2.9%)	9.2% (2004; 8.0–10.5%)	–
Slovakia	13.5% (1584; 11.9–15.3%)	0.8% (1584; 0.4–1.4%)	3.3% (1584; 2.5–4.4%)	0.4% (1585; 0.2–0.9%)	–
Slovenia	13.1% (731; 10.8–15.8%)	0.4% (731; 0.1–1.2%)	7.4% (731; 5.6–9.5%)	0.1% (732; 0.0–0.8%)	–
Spain	2.4% (39,526; 2.3–2.6%)	3.1% (39,526; 3.0–3.3%)	0.2% (39,526; 0.2–0.3%)	1.9% (44,559; 1.8–2.1%)	10.7% (98,737; 10.5–10.9%)
Sweden	12.7% (10,046; 12.1–13.4%)	0.6% (10,047; 0.5–0.8%)	13.3% (10,050; 12.7–14.0%)	0.1% (10,050; 0.1–0.2%)	–
Switzerland	4.7% (1006; 3.5–6.2%)	3.1% (1006; 2.1–4.3%)	7.5% (1006; 5.9–9.3%)	2.0% (1013; 1.2–3.0%)	12.2% (221; 8.2–17.3%)
United Kingdom	1.2% (2631; 0.8–1.7%)	1.4% (2631; 1.0–1.9%)	1.1% (2631; 0.8–1.6%)	1.1% (2774; 0.8–1.6%)	–

**Table 4:** Rate of co-positives vs expected rate by chance for all results within the study period 2016–2020

Pathogen 1	Pathogen 2	Positive pathogen 1 (%)	Positive pathogen 2 (%)	Expected co-positivity (%)	Co-positivity (%)	Total *n*	Chi-square
*Anaplasma *spp.	*Ehrlichia *spp.	6.19	3.82	0.24	0.81	224,023	*P *< 0.001*
*Anaplasma *spp.	*Dirofilaria immitis*	6.19	1.89	0.12	0.17	224,022	*P *< 0.001*
*Anaplasma *spp.	*Leishmania *spp.	3.19	9.71	0.31	0.78	53,505	*P *< 0.001*
*Anaplasma *spp.	*Borrelia burgdorferi*	6.19	2.69	0.17	0.50	224,022	*P *< 0.001*
*Ehrlichia *spp.	*Dirofilaria immitis*	3.82	1.89	0.07	0.22	224,022	*P *< 0.001*
*Ehrlichia *spp.	*Leishmania *spp.	5.44	9.71	0.53	1.44	53,504	*P *< 0.001*
*Ehrlichia *spp.	*Borrelia burgdorferi*	3.82	2.69	0.10	0.10	224,024	*P *= 0.657
*Dirofilaria immitis*	*Leishmania *spp.	1.39	9.17	0.13	0.30	57,953	*P *< 0.001*
*Dirofilaria immitis*	*Borrelia burgdorferi*	1.89	2.69	0.05	0.06	224,030	*P *= 0.083
*Leishmania *spp.	*Borreliaburgdorferi*	9.71	0.18	0.02	0.04	53,504	*P *< 0.001*

Vector commonalities or endemic rates within geographies was not included in the comparison

*Statistically significant

## Abbreviations

CVBD: Canine vector-borne disease
IFA: Immunofluorescense assay
qELISA: Quantitative enzyme linked immunosorbent assay

## Footnotes

1 R Core Team (2020). R: A language and environment for statistical computing. R Foundation for Statistical Computing, Vienna, Austria. https://www.R-project.org/.

2 Wickham, H. 2021. https://CRAN.R-project.org/package=tidyverse.

3 Lahti, L et al. 2021. Tools for Eurostat Open Data. https://CRAN.R-project.org/package=eurostat.

4 https://ec.europa.eu/eurostat/web/nuts/background.
